# CLASP Mediates Microtubule Repair by Restricting Lattice Damage and Regulating Tubulin Incorporation

**DOI:** 10.1016/j.cub.2020.03.070

**Published:** 2020-06-08

**Authors:** Amol Aher, Dipti Rai, Laura Schaedel, Jeremie Gaillard, Karin John, Qingyang Liu, Maarten Altelaar, Laurent Blanchoin, Manuel Thery, Anna Akhmanova

**Affiliations:** 1 Cell Biology, Neurobiology and Biophysics, Department of Biology, Faculty of Science, Utrecht University, Padualaan 8, 3584 CH Utrecht, the Netherlands; 2 University of Grenoble-Alpes, CEA, CNRS, INRA, Interdisciplinary Research Institute of Grenoble, Laboratoire de Phyiologie Cellulaire & Végétale, CytoMorpho Lab, 38054 Grenoble, France; 3 University of Grenoble-Alpes, CNRS, Laboratoire Interdisciplinaire de Physique, 38000 Grenoble, France; 4 Biomolecular Mass Spectrometry and Proteomics, Bijvoet Center for Biomolecular Research, Utrecht Institute for Pharmaceutical Sciences and the Netherlands Proteomics Centre, Utrecht University, Padualaan 8, 3584 CH Utrecht, the Netherlands; 5 Université de Paris, INSERM, CEA, Institut de Recherche Saint Louis, U 976, CytoMorpho Lab, 75010 Paris, France

**Keywords:** microtubule dynamics, microtubule catastrophe, microtubule rescue, microtubule repair, lattice defects, tubulin, CLASP, TOG2, laser microsurgery, *in vitro* reconstitution

## Abstract

Microtubules play a key role in cell division, motility, and intracellular trafficking. Microtubule lattices are generally regarded as stable structures that undergo turnover through dynamic instability of their ends [1]. However, recent evidence suggests that microtubules also exchange tubulin dimers at the sites of lattice defects, which can be induced by mechanical stress, severing enzymes, or occur spontaneously during polymerization [2, 3, 4, 5, 6]. Tubulin incorporation can restore microtubule integrity; moreover, “islands” of freshly incorporated GTP-tubulin can inhibit microtubule disassembly and promote rescues [3, 4, 6, 7, 8]. Microtubule repair occurs *in vitro* in the presence of tubulin alone [2, 3, 4, 5, 6, 9]. However, in cells, it is likely to be regulated by specific factors, the nature of which is currently unknown. CLASPs are interesting candidates for microtubule repair because they induce microtubule nucleation, stimulate rescue, and suppress catastrophes by stabilizing incomplete growing plus ends with lagging protofilaments and promoting their conversion into complete ones [10, 11, 12, 13, 14, 15, 16, 17]. Here, we used *in vitro* reconstitution assays combined with laser microsurgery and microfluidics to show that CLASP2α indeed stimulates microtubule lattice repair. CLASP2α promoted tubulin incorporation into damaged lattice sites, thereby restoring microtubule integrity. Furthermore, it induced the formation of complete tubes from partial protofilament assemblies and inhibited microtubule softening caused by hydrodynamic-flow-induced bending. The catastrophe-suppressing domain of CLASP2α, TOG2, combined with a microtubule-tethering region, was sufficient to stimulate microtubule repair, suggesting that catastrophe suppression and lattice repair are mechanistically similar. Our results suggest that the cellular machinery controlling microtubule nucleation and growth can also help to maintain microtubule integrity.

## Highlights

• CLASP stabilizes damaged microtubule lattices
• CLASP converts partial protofilament assemblies into complete tubes
• CLASP promotes complete repair of microtubule lattice defects
• CLASP inhibits softening of microtubules bent by hydrodynamic flow

## Graphical Abstract

**Figure:**
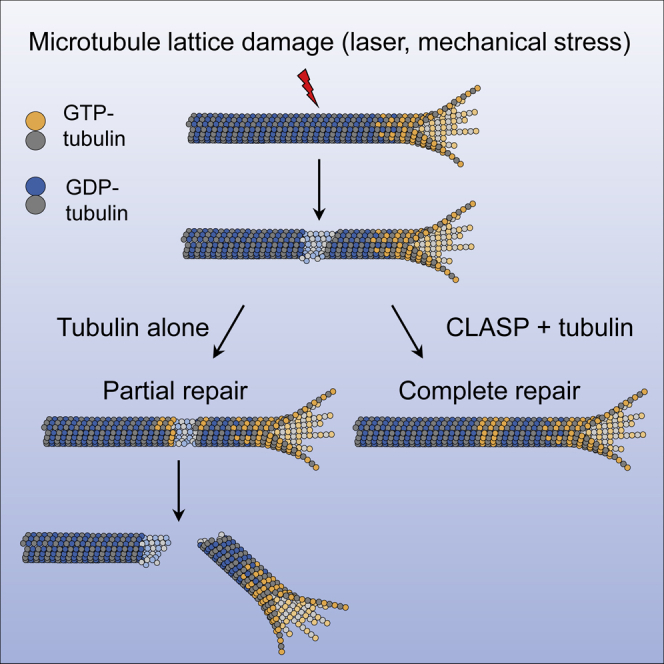

## Teaser

Using *in vitro* reconstitution assays combined with laser microsurgery and microfluidics, Aher et al. show that CLASP, a positive regulator of microtubule growth, promotes microtubule repair by stabilizing incomplete lattices and stimulating continuous tubulin incorporation into damaged sites.

## Results and Discussion

### CLASP Stalls Depolymerization and Promotes Repair of Microtubule Lattices Damaged by Photoablation

**Figure 1:**
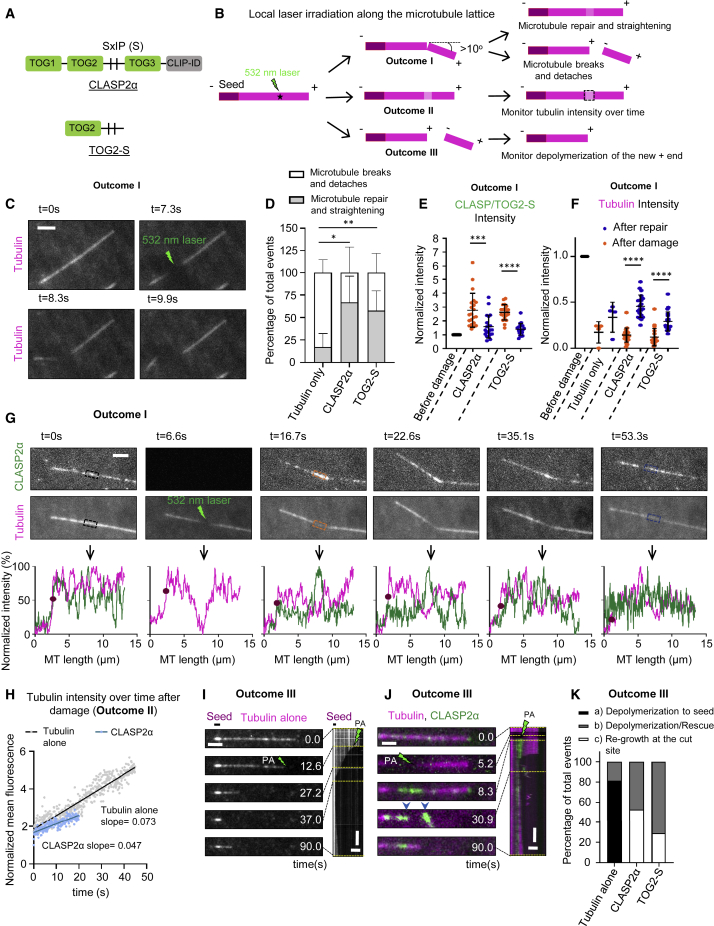
CLASP Promotes Repair of Microtubule Lattices Damaged by Laser Illumination (A) A scheme of full-length CLASP2α and its TOG2-S fragment. Vertical lines labeled SxIP (Ser-any amino acid-Ile-Pro) represent EB-binding motifs located in the unstructured positively charged region adjacent to the TOG2 domain. (B) Schematic for an experiment to monitor the possible outcomes of a 532 nm pulsed laser induced damage at a site along the dynamic lattice. (I) Microtubule bending at the site of damage, which can lead to either straightening of the lattice or microtubule breakage. (II) Reduction of the tubulin intensity. (III) Microtubule severing resulting in direct appearance of two microtubule ends. (C) Stills from a time-lapse video showing photodamage of a dynamic microtubule grown in the presence of Rhodamine-tubulin alone followed by bending and subsequent breakage (outcome I). Scale bar, 2 μm. (D) Percentage of total events for outcome I resulting in either microtubule breakage or straightening at the point of photodamage in the presence of tubulin alone (n = 22 microtubules analyzed from 4 experiments) or together with either 30 nM GFP-CLASP2α (n = 53 microtubule analyzed from 6 experiments) or 30 nM GFP-TOG2-S (n = 54 from 8 experiments). Error bars denote SD. (E and F) Normalized mean intensity at the site of photodamage in case of outcome I for the GFP channel for CLASP2α and TOG2-S (E) and Rhodamine-tubulin channel in the presence of tubulin alone or together with either CLASP2α or TOG2-S (F); before damage (black), immediately after damage (orange) and after microtubule straightening (blue). Tubulin alone: n = 4 microtubules, 4 experiments; CLASP2α: n = 21 microtubules, 4 experiments; TOG2-S: n = 20 microtubules, 6 experiments. Error bars denote SD. (G) Stills from a time-lapse video showing a dynamic microtubule grown in the presence of Rhodamine-tubulin together with 30 nM GFP-CLASP2α for outcome I. Normalized intensity profiles along the microtubule for the CLASP (green) and tubulin channel (magenta) at different time points are shown in the bottom panels, with the arrow pointing to the site of photodamage. The purple circle on the plot indicates the end of the microtubule. Scale bars, 2 μm. (H) Normalized mean tubulin fluorescence intensity over time at the site of local photodamage (outcome II); microtubules were grown in the presence of Rhodamine tubulin alone (gray) (n = 35 microtubules, 2 experiments) or together with 30 nM GFP-CLASP2α (blue) (n = 44 microtubules, 2 experiments). Straight lines were fitted to the initial increase in tubulin intensity until saturation for the respective mean values yielding slopes as indicated. (I and J) Stills and the corresponding kymograph of a microtubule grown in the presence of Rhodamine-tubulin alone (I) or together with GFP-CLASP2α (30 nM) (J) severed with a 532 nm laser as indicated (outcome III). Scale bars: still image, 2 μm; kymograph, 4 μm (horizontal) and 10 s (vertical). Dotted yellow lines point to the time point of the still in the kymograph. (K) Percentage of total laser severing events resulting in either immediate microtubule regrowth at the site of photoablation, microtubule depolymerization to the seed or depolymerization followed by rescue along the lattice, in the presence of Rhodamine-tubulin alone or together with either 30 nM GFP-CLASP2α or 30 nM GFP-TOG2-S. Tubulin alone: n = 186 microtubules, 3 experiments: CLASP2α: n = 36 microtubules, 3 experiments; TOG2-S: n = 48 microtubules, 8 experiments. For plots in Figure 1D: ^∗^p = 0.0091, ^∗∗^p = 0.0381, for Figures 1E and 1F, ^∗∗∗∗^p < 0.0001, ^∗∗∗^p = 0.001 Mann-Whitney U test. See also Figures S1 and S2, Table S1, and Videos S1 and S2.

To investigate whether CLASPs can promote microtubule repair, we modified previously described *in vitro* reconstitution assays with GFP-tagged CLASP2α purified from HEK293T cells (Figure S1A; Table S1) [12]. Microtubules were grown from GMPCPP-stabilized seeds, visualized by adding fluorescently labeled tubulin and observed by total internal reflection fluorescence (TIRF) microscopy [12, 18]. In this assay, GFP-CLASP2α (Figure 1A) shows some binding to microtubule lattices and a weak enrichment at growing microtubule tips [12]. To explore the capacity of CLASP to repair damaged microtubules, we performed laser-mediated microsurgery on dynamic microtubules. Laser irradiation at a point along microtubule lattice resulted in local reduction of the tubulin intensity with or without microtubule bending (outcomes I and II), or lattice severing, distinguished by the appearance of two microtubule ends at the irradiated site (outcome III) (Figure 1B). In the presence of tubulin alone, microtubules that bent by more than 10° after damage (outcome I) typically broke (Figures 1B and 1C; Video S1), although in 18% of the cases, microtubules straightened again, suggesting that they were repaired (Figure 1D). These data are in agreement with previous work showing that damaged microtubule lattices can be autonomously repaired by tubulin incorporation [2, 3, 6, 9]. When these experiments were performed in the presence of CLASP2α, the percentage of successful repair of microtubules bent at an irradiated site by more than 10° increased 3-fold to 62% (Figures 1D and 1G).

Video S1. CLASP Promotes Repair of Microtubule Lattices Damaged by Laser Illumination, Related to Figure 1 Microtubule lattice repair at the laser induced damage site in a microtubule grown in the presence of Rhodamine-tubulin alone (upper panel, corresponds to Figure 1C), a microtubule grown in the presence of Rhodamine-tubulin (magenta) and 30 nM GFP-CLASP2α (green) (middle panel, corresponds to Figure 1G) and a microtubule grown in the presence of Rhodamine-tubulin (magenta) and 30 nM GFP-TOG2-S (green) (lowermost panel, corresponds to Figure S1B). Yellow arrowheads point to the site of laser induced photodamage. Images were acquired using a TIRF microscope in a stream mode at a 100 ms interval. Video is sped up 50 times. Time is shown in seconds.

Mammalian CLASPs contain three TOG-like domains, TOG1, TOG2, and TOG3, connected by flexible positively charged linkers, and a C-terminal domain (CLIP-ID) that binds to different partners and targets CLASPs to various subcellular locations [17, 19] (Figure 1A). Our previous work has shown that an isolated TOG2 domain has a very low affinity for microtubules and does not bind to free tubulin [12]. However, when TOG2 was fused to the adjacent intrinsically disordered positively charged region (a fusion protein termed TOG2-S, Figure 1A), it could bind to microtubule lattice, show some autonomous enrichment at growing microtubule ends, and suppress catastrophes even in the absence of end-binding (EB) proteins, which normally target CLASPs to growing microtubule plus ends [12]. By performing laser damage experiments in the presence of TOG2-S, we found that it could also promote repair and straightening of microtubules bent after irradiation (Figures 1D, S1A, and S1B; Video S1). Although mass spectrometry analysis of CLASP2α revealed the presence of some proteins binding to CLASP C terminus, such as CLIP-170 (Table S1), the repair activity observed for TOG2-S, which lacks the partner-binding C-terminal region of CLASP, indicates that the ability to promote microtubule restoration is autonomous to CLASP.

Before irradiation, CLASP2α weakly labeled entire microtubules, and its fluorescence intensity was the same at the sites that were subsequently repaired and the sites that broke (Figure S1C). However, after irradiation, both CLASP2α and TOG2-S rapidly bound to the sites of damage (Figures 1E, 1G, S1B, S1D, and S1E), indicating they can autonomously recognize such sites. Over time, the accumulation of CLASP2α and TOG2-S diminished, whereas tubulin intensity at the irradiated site increased (Figures 1E–1G, S1B, S1D, S1E, and S1G; Video S1). Such an increase was also observed with tubulin alone (Figures 1F and S1F). However, since repair of bent microtubules in the absence of CLASP2α or TOG2-S was infrequent, we could not reliably compare tubulin incorporation rates with and without CLASP. To obtain a larger number of measurements, we monitored microtubules that did not bend but displayed reduced tubulin intensity at the site of damage, likely due to a combination of photobleaching and loss of tubulin dimers (Figure 1B, outcome II). We found that both with tubulin alone and in the presence of CLASP2α, tubulin intensity at the damaged sites first increased linearly after irradiation and then reached a plateau (Figures S1H–S1J). The rate of tubulin incorporation based on the slopes of the linear part of the plot was ∼1.7-fold higher for tubulin alone compared to the condition where CLASP2α was included in the assay (Figure 1H), and the tubulin intensity after repair at the irradiated sites with respect to the intensity immediately after damage increased ∼5.4-fold in the case of tubulin alone but only ∼1.9-fold in the presence of CLASP2α (Figures S1H–S1J). Since bent microtubules were restored more often in the presence of CLASP2α compared to tubulin alone (Figure 1D), this suggests that CLASP2α most likely promotes rapid repair by inhibiting microtubule disassembly at the irradiated site and thus limits the zone where new tubulin can incorporate.

We next tested whether CLASP2α had a stabilizing effect on microtubule ends generated upon complete microtubule severing (outcome III) (Figure 1B). In agreement with previous work [20], we found that in the presence of tubulin alone, 81% of freshly severed microtubule plus ends depolymerized to the seed, whereas the remaining 19% were rescued along the lattice (Figures 1I–1K; Video S2). In the presence of CLASP2α, the depolymerization of newly generated plus ends was strongly inhibited: 53% of the microtubules promptly re-grew directly from the ablation site (Figures 1J and 1K; Video S2). The remaining 47% were rescued along the dynamic lattice, in line with the fact that CLASPs act as rescue factors [10, 11, 12, 13, 21] (Figure 1K). TOG2-S fusion was also sufficient to suppress depolymerization of severed plus ends and promoted re-growth at the site of photo-ablation in 29% of the cases, although the protection was less efficient than with full-length CLASP2α (Figures 1K and S2A). Importantly, in the presence of either CLASP2α or TOG2-S, none of the ablated microtubules depolymerized to the seed (Figure 1K), and most of these microtubules exhibited only very short depolymerization excursions (<1 μm) compared to tubulin alone (Figure S2B). We note that observation of depolymerization events in the presence of CLASP2α or TOG2-S was not limited by the lengths of the seed-proximal microtubule parts after severing, which were longer than the depolymerization lengths (Figure S2C).

Video S2. CLASP Prevents Depolymerization of Laser-Severed Microtubules, Related to Figure 1 Microtubules were ablated with a laser in the presence of Rhodamine-tubulin alone (upper panel, corresponds to Figure 1I), in the presence of Rhodamine-tubulin (magenta) and 30 nM GFP-CLASP2α (green) (middle panel, corresponds to Figure 1J) or in the presence of Rhodamine- tubulin (magenta) and 30 nM GFP-TOG2-S (green) (lower panel, corresponds to Figure S2A). Yellow asterisks indicate the site of laser severing and the yellow arrowheads indicate the position of microtubule breakage. Images were acquired using a TIRF microscope in a stream mode at a 100 ms interval. Video is sped up 90 times. Time is shown in seconds.

We also tested whether CLASP2α could protect freshly generated microtubule ends in the absence of tubulin. To test this, we performed local laser ablation of microtubules in the absence of free tubulin, which were capped with GMPCPP-tubulin to prevent their depolymerization (Figure S2D). All freshly generated microtubule ends depolymerized to the seed or the GMPCPP cap in the absence of CLASP2α but were protected in its presence (Figures S2E–S2H). We conclude that CLASP2α promotes microtubule repair, and this activity is at least partly dependent on recognition of damaged microtubule lattices and inhibition of their disassembly.

### CLASP Converts Partial Protofilament Assemblies into Complete Tubes

**Figure 2:**
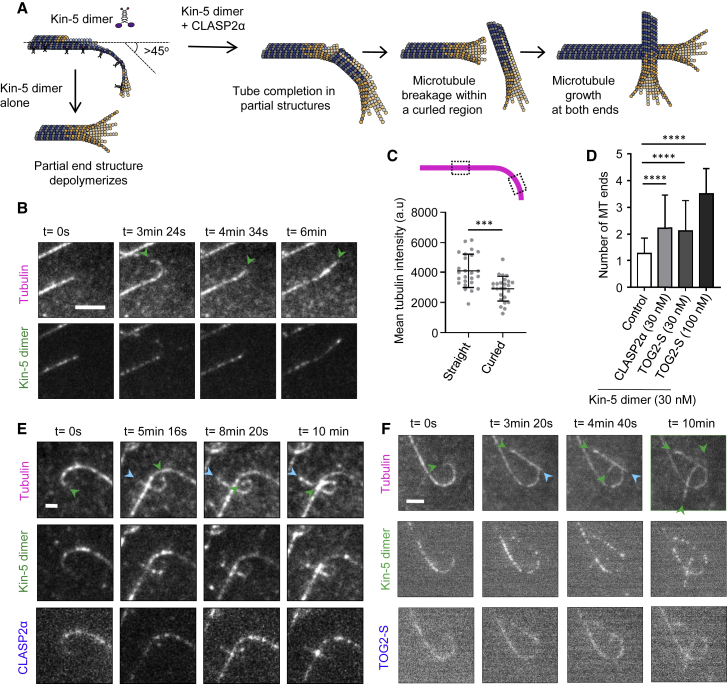
CLASP Promotes Formation of Complete Microtubules from Partial Protofilament Assemblies (A) Cartoon illustrating the changes in tubulin sheet- or ribbon-like structures generated at the plus ends of dynamic microtubules in the presence of 30 nM kinesin-5-GFP dimer (Kin-5 dimer) alone or together with 30 nM GFP-CLASP2α. (B) Stills from a time-lapse video showing a plus end of a microtubule grown in the presence of Rhodamine-tubulin and 30 nM Kin-5-GFP dimer. Scale bar: 2 μm. (C) Mean tubulin intensity values for the straight and the curled portions of the microtubule lattice as indicated for microtubules grown in the presence of Rhodamine tubulin and 30 nM Kin-5-GFP dimer. n = 25 microtubules from 2 experiments. Error bars denote SD. (D) Number of newly generated microtubule ends from a single microtubule plus end for microtubules grown in the presence of Rhodamine-tubulin and 30 nM Kin-5-GFP dimer alone (n = 38 microtubule plus ends, 3 experiments) or together with 30 nM TagBFP-CLASP2α (n = 95 microtubule plus ends, 3 experiments), or with 30 nM TagBFP-TOG2-S (n = 85 microtubule plus ends, 3 experiments), or with 100 nM Tag-BFP-TOG2-S (n = 26 microtubule plus ends, 2 experiments). Events where the microtubule plus ends bent by angles over 45° with respect to the lattice were monitored in a 10 min time lapse. Error bars denote SD. (E) Stills from a time-lapse video showing the plus end for a microtubule grown in the presence of Rhodamine tubulin, 30 nM Kin-5-GFP dimer, and 30 nM TagBFP-CLASP2α. Scale bar: 2 μm. (F) Stills from a time-lapse video showing a microtubule plus end grown in the presence of Rhodamine-tubulin, 30 nM Kin-5-GFP dimer together with 30 nM TagBFP-TOG2-S. Scale bar: 3 μm. For (B), (E), and (F), green arrowheads point to the plus end and blue arrowheads to the minus ends. For all plots, ^∗∗∗∗^p < 0.0001, ^∗∗∗^p = 0.001 and ns, no significant difference with control, Mann-Whitney U test. See also Video S3.

The experiments described above suggest that CLASP promotes formation of complete tubes from microtubules lacking some parts of protofilaments. To further test the effect of CLASP on partial protofilament assemblies, we used a previously described engineered kinesin-5 dimer, which consists of the motor domain and neck linker of *Xenopus* kinesin-5 (Eg5) fused to the motor-proximal coiled coil derived from *Drosophila* kinesin-1 [22]. This engineered kinesin-5 (Kin-5 dimer) was shown to generate long tubulin ribbons and protofilament sheets at microtubule plus ends [22] (Figure 2A), likely by stabilizing specific tubulin conformations and enhancing lateral contacts between tubulin dimers [23]. In the presence of Kin-5 dimer, we observed curled microtubule ends, which displayed a 25% reduction in tubulin intensity compared to the straight parts of the lattice, in line with the idea that the curled regions miss some protofilaments (Figures 2B and 2C). Similar curved microtubule plus-end extensions were also formed when CLASP2α or TOG2-S were included with the Kin-5 dimer in the assay (Figures 2A, 2E, and 2F; Video S3). However, whereas in the presence of the Kin-5 dimer alone these structures were transient and typically depolymerized, in the presence of CLASP2α or TOG2-S, curled microtubule ends were converted into multiple straight microtubules (Figures 2D–2F). These data suggest that CLASP2α and its TOG2-S fragment can promote formation of complete microtubules from protofilament sheets. Since several microtubules could form from a single curled end, these results suggest that CLASP2α and TOG2-S not only help to repair strongly tapered microtubule ends, as it occurs during catastrophe suppression [12] but also likely promote extension of protofilament bundles from the side, allowing them to close into complete tubes.

Video S3. CLASP Promotes Formation of Complete Microtubules from Partial Protofilament Assemblies, Related to Figure 2 Microtubules were grown in the presence of Rhodamine-tubulin (magenta) and 30 nM Kin-5-GFP dimer (green) (upper left panel, corresponds to Figure 2B), in the presence of Rhodamine-tubulin (magenta), 30 nM Kin-5-GFP (green) and 30 nM TagBFP-CLASP2α (blue) (upper right panel, corresponds to Figure 2E), in the presence of Rhodamine-tubulin (magenta), 30 nM Kin-5-GFP (green) and 30 nM TagBFP-TOG2-S (blue) (bottom left panel, corresponds to Figure 2F) or in the presence of Rhodamine-tubulin (magenta), 30 nM Kin-5-GFP (green) and 100 nM TagBFP-TOG2-S (blue) (lower-right panel). Yellow arrowheads indicate the position of microtubule curling induced by the Kinesin-5 dimer. Images were acquired using a TIRF microscope at a 4 s interval. Video is sped up 10 times. Time is shown in seconds.

### CLASP Promotes Complete Repair of Microtubule Lattice Defects

**Figure 3:**
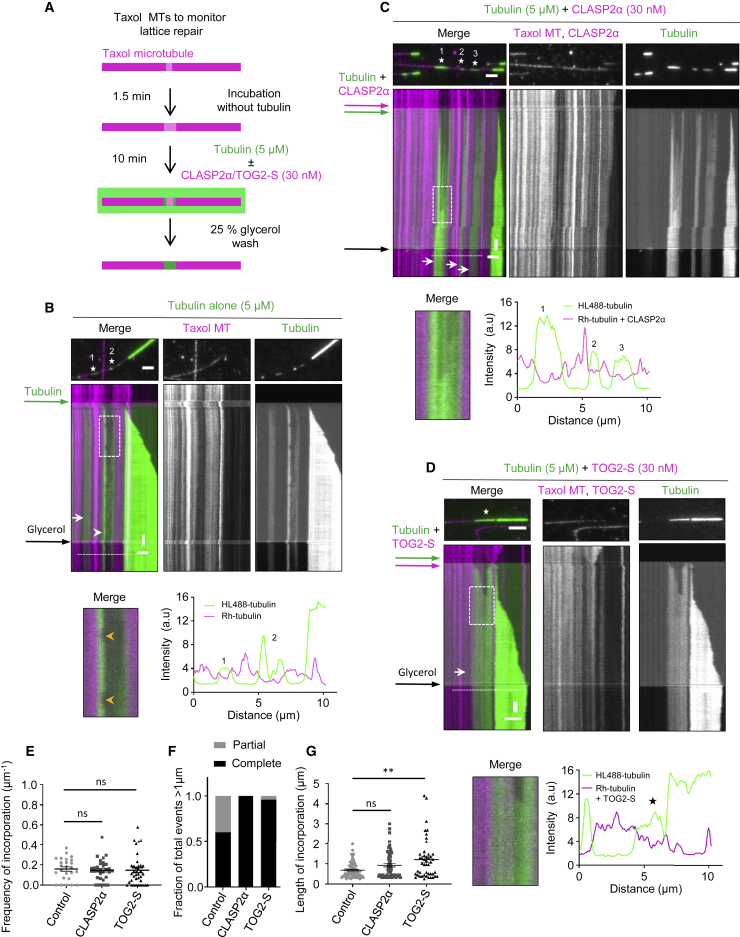
CLASP Promotes Complete Repair of Damaged Microtubule Lattices (A) Schematic for an experiment to monitor tubulin incorporation into damaged microtubule lattices. Microtubules prepared from Rhodamine-tubulin in the presence of Taxol were first incubated without Taxol and tubulin for 1.5 min and subsequently with 5 μM HiLyte Fluor-488-labeled tubulin with or without 30 nM mCherry-CLASP2α or 30 nM mCherry-TOG2-S. After 10 min, the residual free green tubulin was washed out with the wash buffer supplemented with 25% glycerol to prevent microtubule depolymerization, in order to better visualize incorporation of green tubulin. (B–D) Microtubule repair in the presence of tubulin alone (B) or together with either 30 nM mCherry-CLASP2α (C) or 30 nM mCherry-TOG2-S (D). Single frames (top) of time-lapse videos after the final washout and kymographs (bottom) showing green tubulin incorporation sites (numbered asterisks in stills) into Rhodamine-labeled microtubule lattices (magenta). In kymographs, white arrows indicate complete repair and white arrowheads partial repair. Enlarged views of the boxed regions in the kymographs in (B)–(D) showing partial (B) or complete microtubule repair (C and D) in the bottom-left panel for each condition. Yellow arrowheads in (B) (bottom left) indicate events of loss of freshly incorporated tubulin. Intensity profiles along the microtubule for the Rhodamine-labeled microtubule seed channel (magenta) with or without mCherry-CLASP2α, and for the green tubulin channel are shown in the bottom-right panel for each condition. The numbers indicate incorporation sites specified in stills in (B) and (D). Scale bars: 2 μm (horizontal) and 60 s (vertical). See also Video S4. (E and G) Frequency of incorporation per unit length per microtubule (E) and the average length of incorporations (G) in the presence of tubulin alone (n = 73, M = 25, L = 459.25 μm, 2 experiments), together with 30 nM mCherry-CLASP2α (n = 64, M = 31, L = 450.74 μm, 5 experiments) or 30 nM mCherry-TOG2-S (n = 52, M = 37, L = 418.43 μm, 4 experiments), where n, M, and L are total number of incorporations, total number of microtubules, and total length of microtubules analyzed, respectively. Error bars represent SEM. ^∗∗^p = 0.0038 and ns, no significant difference with control, Mann-Whitney U test. (F) Fraction of total events resulting in either complete or partial repair at the site of tubulin incorporations with the length exceeding 1 μm, in the presence of tubulin alone (n = 15, 2 experiments), together with either 30 nM mCherry-CLASP2α (n = 22, 5 experiments) or 30 nM mCherry-TOG2-S (n = 23, 4 experiments), where n is the total number of incorporations longer than 1 μm. See also Video S4.

To generate a substrate in which we could monitor tubulin incorporation into partially damaged microtubule lattices, we prepared Rhodamine-labeled microtubules stabilized with Taxol. Microtubules polymerized in the presence of Taxol are known to exhibit extensive lattice defects [24]; this property has been used, for example, to demonstrate the impact of microtubule defects on kinesin-based transport [25, 26]. Taxol-stabilized microtubules display more structural defects in the lattice when incubated with very low tubulin concentrations [27]. To promote defect formation, we incubated Taxol-stabilized microtubules for 1.5 min in a buffer without Taxol and tubulin (Figure 3A). In these conditions, microtubules gradually lose tubulin dimers from discrete sites, which can be detected as gaps with a reduced intensity of tubulin signal (Figure 3B; Video S4). When 5 μM tubulin with a green (HiLyte Fluor488) fluorescent label was added to such partially “eroded” Rhodamine-labeled microtubules, we observed that green tubulin was incorporated not only at microtubule ends but also into the microtubule lattices where the original tubulin signal was reduced (Figures 3B; Video S4). The addition of CLASP2α or TOG2-S had no effect on the frequency of tubulin incorporation sites, as they depended on the number of defects induced by Taxol washout and the extent of microtubule erosion (Figures 3C–3E). However, CLASP2α and TOG2-S increased the percentage of damaged lattice sites that appeared completely “healed” (Figure 3F; Video S4). This was because the polymerization of freshly added tubulin within such sites was continuous, whereas, in the presence of tubulin alone, incorporation was often confined to the edges of the gaps (Figures 3B–3D). As a result, the average length of the analyzed incorporation sites appeared slightly longer in the presence of CLASP2α or TOG-S, and, in case of TOG2-S, this difference was significant (Figure 3G). Importantly, in this assay, free tubulin concentration was lower than in the assays described above (5 μM versus 15 μM in Figures 1 and 2). This concentration was chosen to allow tubulin incorporation at the ends or the eroded lattice but prevent nucleation of new microtubules, which was observed when CLASP2α was combined with partially damaged Taxol-stabilized microtubules at high free tubulin concentration and which complicated the analysis. Altogether, our results indicate that CLASP2α or its microtubule-tethered TOG2 domain promotes continuous tubulin addition to the ends of partial protofilament assemblies, allowing efficient repair of lattice gaps.

Video S4. CLASP Promotes Complete Repair of Damaged Microtubule Lattices, Related to Figure 3 Taxol-stabilized microtubules (magenta) were initially incubated in a buffer without Taxol and tubulin for 1.5 min and then transferred into a buffer with either 5 μM HiLyte 488-labeled tubulin alone (green) (upper panel, corresponds to Figure 3B), 5 μM HiLyte 488-labeled tubulin together with 30 nM mCherry-CLASP2α (middle panel, corresponds to Figure 3C) or 5 μM HiLyte 488 labeled tubulin together with 30 nM mCherry-TOG2-S (lower panel, corresponds to Figure 3D) for 10 minutes followed by a 25% glycerol wash. Green tubulin incorporation into the lattice is indicated by numbers for the upper and middle panels and by an asterisk for the lower panel. Images were acquired using a TIRF microscope at a 2 s interval. Video is sped up 75 times. Time is shown in minutes.

**Figure 4:**
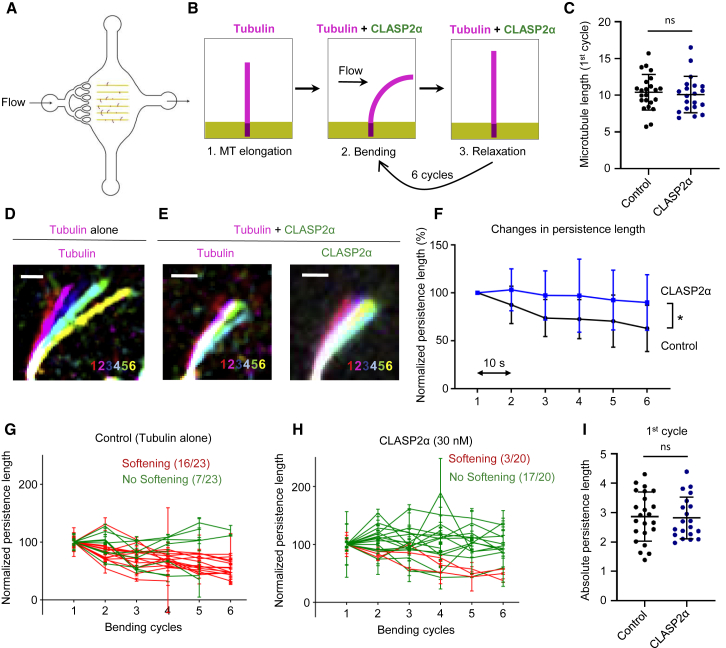
CLASP2α Inhibits Microtubule Softening Induced by Hydrodynamic Flow (A) Illustration of the microfluidic device used for microtubule bending. (B) Scheme of the work sequence: 1. Red fluorescent microtubules were grown from seeds grafted on micropatterned lines. 2. Microtubules were bent for 10 s by applying a fluid flow using the same mix as in 1, with or without 30 nM GFP-CLASP2α. 3. The flow was stopped for 10 s before repeating the bending cycle. (C) Lengths of microtubules used in the bending experiments in the presence of tubulin alone (n = 23 microtubules) or together with 30 nM GFP-CLASP2α (n = 20 microtubules). Error bars denote SD. (D and E) Images showing an overlay of maximum bent conformations where every cycle is represented in a different color for the tubulin channel for a microtubule bent in the presence of tubulin alone (D) and for both tubulin and CLASP channels for a microtubule bent in the presence of tubulin together with 30 nM GFP-CLASP2α (E). Scale bar: 3 μm. (F) Persistence length measured for microtubules bent in the presence (blue curve, n = 20) or absence (black curve, n = 23) of 30 nM GFP-CLASP2α. Persistence length was normalized to the value in the first bending cycle for each microtubule. Values represent the average persistence length (mean ± SD) of individual measurements shown in Figures 4G and 4H. To test whether microtubules showed softening, a Spearman correlation test for persistence length values over subsequent bending cycles was performed. It revealed significant softening of microtubules in both conditions (p = 0.01 and 0.08, respectively), though it was much less pronounced in the presence of CLASP2α. A t test confirmed significant difference between the two curves (p = 0.006). (G and H) Individual persistence length measurements of microtubules bent in the absence (G) and presence (H) of 30 nM GFP-CLASP2α. Values represent the average of five independent measurements for each bending cycle (mean ± SD) and were normalized to the initial value. A Spearman correlation test was performed to test for softening. Red lines indicate microtubules that became significantly softer, and green lines indicate microtubules that did not show significant softening. (I) Absolute persistence lengths for microtubules bent in the presence of tubulin alone (n = 23 microtubules) or together with 30 nM GFP-CLASP2α (n = 20 microtubules) after the 1^st^ cycle. Error bars denote SD. See also Video S5.

Video S5. CLASP2α Inhibits Microtubule Softening Induced by Hydrodynamic Flow, Related to Figure 4 Microtubules (magenta) grown from seeds on micropatterned coverglass bent by orthogonal flow Microtubules were bent for ten seconds by applying a fluid flow with tubulin alone (left panel, corresponds to Figure 4D) or with 30 nM GFP-CLASP2α (right panel, corresponds to Figure 4E). The flow was stopped for ten seconds before repeating the bending cycle. This was repeated for 6 cycles. Scale bar: 3 μm Images were acquired using a TIRF microscope at a 0.3 s interval. Video is sped up 17 times. Time is shown in seconds.

### Microtubules Exhibit Increased Resistance to Mechanical Stress in the Presence of CLASP

Finally, we investigated the effect of CLASP on dynamic microtubules damaged in a more natural way, by inducing mechanical stress with a microfluidics setup described previously [2]. In order to study the impact of CLASP on the deformation of microtubules induced by mechanical forces, we applied cycles of hydrodynamic bending stress to microtubules in the absence or presence of CLASP2α. To this end, we grew dynamic microtubules in the absence of CLASP2α from GMPCPP and Taxol-stabilized seeds attached to micropatterns inside a microfluidic device (Figure 4A). Microtubules were then bent by an orthogonal fluid flow for 10 s in either the absence or presence of CLASP2α. The flow was subsequently stopped for 10 s and the bending cycle was repeated (Figure 4B) [28]. Previous work showed that microtubules bend and subsequently straighten after each flow application, but the degree of softening (quantified by monitoring the persistence length of microtubules) increases with each cycle due to the gradual loss of tubulin dimers from the lattice [2]. In the presence of tubulin alone, 70% of the microtubules showed softening after six bending cycles; quantification of the persistence length of the entire microtubule population showed that on average, they became 37% softer (Figures 4C, 4D, 4F, and 4G). In the presence of CLASP2α, the average drop in the persistence length of microtubules that were of similar length as the control ones (Figure 4C) was much less pronounced (10%), with only 15% of the microtubules showing softening (Figures 4E, 4F, and 4H). This indicates that microtubules exhibit increased resistance to mechanical stress in the presence of CLASP2α. These data support the idea that CLASP either alters the mechanical properties of microtubules or promotes repair of the damage induced by bending. Since we did not observe any difference in the persistence length after the first bending cycle (Figure 4I), it is likely that CLASP does not directly alter lattice rigidity but promotes repair of bent lattices.

### Conclusions

Taken together, our data reveal that CLASP2α is an autonomous microtubule repair factor. Our experiments suggest that CLASP repair activity is a combination of recognition and stabilization of damaged but not yet depolymerizing sites within the microtubule lattice and a more continuous tubulin incorporation at the sites of damage. Since full-length CLASP2α and its microtubule-tethered TOG2 domain were both active in microtubule repair assays, and, since TOG2 is highly conserved between CLASP1 and CLASP2 and is present in all CLASP isoforms [19, 29], this property is likely shared by all mammalian CLASPs. Furthermore, given that previous work showed that the TOG2-S fusion can suppress catastrophes [12], our data suggest that the mechanism of microtubule repair is similar to that of catastrophe suppression. Our previous work has shown that CLASPs can stabilize a microtubule plus end with an incomplete set of protofilaments, thereby promoting its recovery into a complete tube [12]. Furthermore, CLASPs are essential for microtubule nucleation from the Golgi in cells [30] and reduce the kinetic threshold for templated microtubule nucleation *in vitro* [12]. In the current study, we showed that CLASP2α promotes formation of complete tubes from tubulin sheets or ribbons generated by kinesin-5 dimer, structures that resemble microtubule nucleation intermediates [23]. All these activities likely depend on the ability of CLASPs to stabilize partial microtubule structures prone to depolymerization, prevent their catastrophic disassembly, and shift the balance toward their continuous polymerization. Given that the microtubule-tethered TOG2 domain, which might be binding between protofilaments [12], was sufficient to promote microtubule lattice repair, this activity might depend on stabilizing lateral contacts between tubulin dimers.

If CLASPs help to repair microtubules rapidly and efficiently, their loss could potentially lead to accumulation of microtubule damage that is repaired slowly. This might explain why CLASP depletion leads to increased guanosine triphosphate (GTP)-tubulin content and EB accumulation along microtubule shafts, a phenotype that could be restored to control levels by the TOG2 domain of CLASP2 [31]. These observations in cells fit well with our observation of lower extent of damage and less extensive tubulin incorporation at irradiated sites in the presence of CLASP. The strong reduction in the density of microtubule networks observed in CLASP-depleted cells could thus be caused not only by reduced microtubule nucleation and plus-end stability as assumed previously but also by the decreased stability of microtubule lattices, which are repaired less efficiently when CLASPs are absent. An important question for future research is whether and how other components of microtubule polymerization machinery contribute to microtubule repair.

## STAR★Methods

### Key Resources Table

**Table:** 

REAGENT or RESOURCE	SOURCE	IDENTIFIER
**Bacterial and Virus Strains**		

E.coli BL21 (DE3)	Agilent	200131

**Chemicals, Peptides, and Recombinant Proteins**		

cOmplete™, EDTA-free Protease Inhibitor Cocktail	Roche	Cat# 4693116001
Tubulin Porcine	Cytoskeleton	Cat# T240-C
Tubulin Porcine TRITC	Cytoskeleton	Cat# TL590M
Tubulin Porcine HiLyte 488™	Cytoskeleton	Cat# TL488M
Tubulin Porcine HiLyte 647™	Cytoskeleton	Cat# TL670M
Tubulin Porcine (Biotin)	Cytoskeleton	Cat# T333P
GMPCPP	Jena Biosciences	Cat# NU-405L
GTP	Sigma-Aldrich	Cat# G8877
Glucose oxidase	Sigma-Aldrich	Cat# G7141
Catalase	Sigma-Aldrich	Cat# C9322
DTT	Sigma-Aldrich	Cat# R0861
k-casein	Sigma-Aldrich	Cat# C0406
StrepTactin Sepharose High Performance	GE Healthcare	Cat# 28-9355-99
NeutrAvidin	Invitrogen	Cat# A-2666
Taxol	Sigma-Aldrich	Cat# T7402
Polyethyleneimine	Polysciences	Cat# 24765-2
Methyl cellulose, 4000 cp	Sigma-Aldrich	Cat# M0512
d-Desthiobiotin	Sigma-Aldrich	Cat# D1411
Coomassie Brilliant Blue	SERVA	Cat# 17525.02
PLL-PEG-biotin	Susos AG, Switzerland	PLL(20)-g[3.5]-PEG(2)/PEG(3.4)-biotin(50%)
Tubulin bovine	[4]	N/A
Fractogel EMD SO, 650 M,	Merck	Cat# 116882
NHS-ATTO	ATTO Tec	Cat# AD488-35 and AD565-35
NHS-Biotin	Thermo Scientific	N/A
tri-ethoxy-silane-PEG (30 kDa)	Creative PEGWorks	PSB-2014
Hellmanex III	HellmaAnalytics	Cat# 9-307-011-4-507
Taxotere	Sigma	Cat# T1912
SU-8 3050	Chimie Tech Services	Cat# SU8-3050/0.5
trichloro(1H,1H,2H,2H-perfluorooctyl)silane	Sigma	Cat# 448931
StrepII-GFP-CLASP2α 1-1527 (full length)	[12]	N/A
StrepII-mCherry-CLASP2α 1-1527 (full length)	This paper	N/A
StrepII-TagBFP-CLASP2α 1-1527 (full length)	[12]	N/A
StrepII-GFP-CLASP2α 295-813 (TOG2-S)	[12]	N/A
StrepII-mCherry-CLASP2α 295-813 (TOG2-S)	This paper	N/A
StrepII-TagBFP-CLASP2α 295-813 (TOG2-S)	[12]	N/A
Kin-5-GFP	[22]	N/A

**Experimental Models: Cell Lines**		

Human: HEK293T	ATCC	CRL-11268

**Recombinant DNA**		

Kin-5-GFP	Dr. William O. Hancock [22]	N/A
StrepII-GFP-CLASP2α 1-1527 (full length)	[12]	N/A
StrepII-mCherry-CLASP2α 1-1527 (full length)	This paper	N/A
StrepII-TagBFP-CLASP2α 1-1527 (full length)	[12]	N/A
StrepII-GFP-CLASP2α 295-813 (TOG2-S)	[12]	N/A
StrepII-mCherry-CLASP2α 295-813 (TOG2-S)	This paper	N/A
StrepII-TagBFP-CLASP2α 295-813 (TOG2-S)	[12]	N/A

**Software and Algorithms**		

ImageJ	NIH	https://imagej.nih.gov/ij/
Metamorph	Molecular Devices	https://www.moleculardevices.com/products/cellular-imaging-systems/acquisition-and-analysis-software/metamorph-microscopy
GraphPad Prism	GraphPad	https://www.graphpad.com/scientific-software/prism/
KymoResliceWide plugin	Eugene Katrukha	https://github.com/ekatrukha/KymoResliceWide

### Lead Contact and Materials Availability

Further information and requests for resources and reagents should be directed to and will be fulfilled by the Lead Contact, Anna Akhmanova (a.akhmanova@uu.nl). Plasmids generated in this study are available upon request.

### Experimental Model and Subject Details

*E.coli* expression strain BL21 (DE3) was used for recombinant expression and purification of Kin-5-GFP. *E.coli* cells were cultured in standard LB medium supplemented with appropriate antibiotics at 37°C. HEK293T cells were used for expression and purification of CLASP2α full-length protein and its truncations. CLASP2α full-length and its truncation constructs were made in modified pEGFP-C1, pmCherry-C1 or pTagBFP-C1 vectors with a StrepII tag as described previously [12]. HEK293T cells were cultured in complete growth medium containing 45% DMEM (Cat# BE12-604F/U1, Lonza), 45% Ham’s F10 (Cat# BE12-618F Lonza), and 10% fetal calf serum (Lonza) supplemented with penicillin and streptomycin (Merck) and maintained in an incubator at 5% CO_2_ level and 37°C temperature. HEK293T cell lines used were not found in the database of commonly misidentified cell lines, maintained by ICLAC and NCBI BioSample, were not authenticated and were negative for mycoplasma contamination.

### Method Details

#### Protein purification for *in vitro* reconstitutions

GFP-CLASP2α, GFP-TOG2-S, mCherry-CLASP2α, mCherry-TOG2-S, Tag-BFP-CLASP2α and Tag-BFP-TOG2-S used in the *in vitro* reconstitutions assays were purified from HEK293T cells using the Strep(II)-streptactin affinity purification as described previously (Figure S1A) [12]. Cells were transfected with the Strep-tagged constructs using polyethylenimine (PEI, Polysciences), in a ratio of 3:1 for PEI:DNA. Cells were harvested 2 days after transfection. Cells from a 15 cm dish were lysed in 500 μl of lysis buffer (50 mM HEPES, 300 mM NaCl and 0.5% Triton X-100, pH 7.4) supplemented with protease inhibitors (Roche) on ice for 15 minutes. The supernatant obtained from the cell lysate after centrifugation at 21,000 × g for 20 minutes was incubated with 40 μl of StrepTactin Sepharose beads (GE) for 45 minutes. The beads were washed 3 times in the lysis buffer without the protease inhibitors. The protein was eluted with 40 μl of elution buffer (50 mM HEPES, 150 mM NaCl, 1 mM MgCl_2_, 1 mM EGTA, 1 mM dithiothreitol (DTT), 2.5 mM d-Desthiobiotin and 0.05% Triton X-100, pH 7.4). Purified proteins were snap-frozen and stored at −80°C. Kin-5-GFP was purified from *E. coli* BL-21 as described before (Figure S1A) [22].

#### *In vitro* microtubule dynamics assays

Reconstitution of microtubule growth dynamics *in vitro* was performed as described previously [12]. GMPCPP-stabilized microtubule seeds (70% unlabeled tubulin, 18% biotin tubulin and 12% of Rhodamine-tubulin or HiLyte 488 tubulin) were prepared as described before [32]. Briefly, the tubulin mix above was incubated at 37°C for 30 minutes at a total tubulin concentration of 20 μM with 1 mM GMPCPP. Microtubules were pelleted in an Airfuge by centrifugation at 119,000 x g for 5 minutes. Subsequently, the microtubules were depolymerized by incubation on ice for 20 minutes. A second cycle of polymerization in the presence of 1 mM GMPCPP was performed at 37°C for 30 minutes. Microtubule seeds were then pelleted as above and diluted in MRB80 buffer (80 mM piperazine-*N*,*N*[prime]-bis(2-ethanesulfonic acid (PIPES)), pH 6.8, supplemented with 10% glycerol and snap frozen in liquid nitrogen and stored in a −80°C freezer until use.

Flow chambers, assembled from plasma-cleaned glass coverslips and microscopic slides were functionalized by sequential incubation with 0.2 mg/ml PLL-PEG-biotin (Susos AG, Switzerland) and 1 mg/ml NeutrAvidin (Invitrogen) in MRB80 buffer (80 mM piperazine-*N*,*N*[prime]-bis(2-ethanesulfonic acid (PIPES)), pH 6.8, supplemented with 4 mM MgCl_2_, and 1 mM EGTA). Microtubule seeds were attached to the coverslip through biotin-NeutrAvidin interactions. Flow chambers were further blocked with 1 mg/ml κ-casein. The reaction mixture with or without CLASP proteins (MRB80 buffer supplemented with 14.5 μM porcine brain tubulin, 0.5 μM Rhodamine-tubulin, 50 mM KCl, 1 mM guanosine triphosphate (GTP), 0.5 mg/ml κ-casein, 0.1% methylcellulose, and oxygen scavenger mix (50 mM glucose, 400 μg/ ml glucose oxidase, 200 μg/ml catalase, and 4 mM DTT)) was added to the flow chamber after centrifugation in an Airfuge for 5 minutes at 119,000 × *g*. For experiments with the Kin-5-GFP, KCl was excluded from the reaction mixture. The flow chamber was sealed with vacuum grease, and dynamic microtubules were imaged immediately at 30°C using TIRF microscopy. All tubulin products were from Cytoskeleton Inc.

#### TIRF microscopy

*In vitro* reconstitution assays were imaged on a TIRF microscope setup as described previously [32] or on an iLas2 TIRF setup (see below). In brief, we used an inverted research microscope Nikon Eclipse Ti-E (Nikon) with the perfect focus system (Nikon), equipped with Nikon CFI Apo TIRF 100x 1.49 N.A. oil objective (Nikon) and controlled with MetaMorph 7.7.5 software (Molecular Devices). The microscope was equipped with TIRF-E motorized TIRF illuminator modified by Roper Scientific France/PICT-IBiSA, Institut Curie. To keep the *in vitro* samples at 30°C, a stage top incubator model INUBG2E-ZILCS (Tokai Hit) was used. For excitation, 491 nm 100 mW Calypso (Cobolt) and 561 nm 100 mW Jive (Cobolt) lasers were used. We used ET-GFP 49002 filter set (Chroma) for imaging of proteins tagged with GFP or ET-mCherry 49008 filter set (Chroma) for imaging of proteins tagged with mCherry. Fluorescence was detected using an EMCCD Evolve 512 camera (Roper Scientific) with the intermediate lens 2.5X (Nikon C mount adaptor 2.5X) or using the CoolSNAP HQ2 CCD camera (Roper Scientific) without an additional lens. In both cases the final magnification was 0.063 μm/pixel.

The iLas2 system (Roper Scientific) is a dual laser illuminator for azimuthal spinning TIRF (or Hilo) illumination and with a custom modification for targeted photomanipulation. This system was installed on Nikon Ti microscope (with the perfect focus system, Nikon), equipped with 150 mW 488 nm laser and 100 mW 561 nm laser, 49002 and 49008 Chroma filter sets, EMCCD Evolve mono FW DELTA 512x512 camera (Roper Scientific) with the intermediate lens 2.5X (Nikon C mount adaptor 2.5X), CCD camera CoolSNAP MYO M-USB-14-AC (Roper Scientific) and controlled with MetaMorph 7.8.8 software (Molecular Device). To keep the *in vitro* samples at 30°C, a stage top incubator model INUBG2E-ZILCS (Tokai Hit) was used. The final resolution using EMCCD camera was 0.065 μm/pixel, using CCD camera it was 0.045 μm/pixel.

Both microscopes were equipped with an iLas system (Roper Scientific France/PICT-IBiSA) for FRAP and photoablation. The 532 nm Q-switched pulsed laser (Teem Photonics) was used for photoablation by targeting the laser on the microtubule lattice on the TIRF microscope and next to the lattice to induce damage. For severing or damage, a 20 by 20 pixel box was used for illumination at 10%–15% laser power of the 532 nm pulsed laser with a maximum of 20 Hz at 100% for 100 ms. For severing, the laser was aimed at a point on the microtubule lattice whereas for damage the laser irradiation was performed very close to the microtubule lattice but not directly at the lattice.

#### Intensity quantifications along the lattice at damage site

For tubulin and CLASP2α/TOG2-S intensity analysis in Figures 1E and 1F, 6 pixel wide lines were manually drawn at the damage site and the mean intensity values obtained from by Fiji were background subtracted. For both the tubulin and CLASP/TOG2-S intensity analysis, the mean intensities before damage, after damage and after repair I(x) were normalized by the mean intensity before damage *I*_*beforedamage*_ yielding mean intensity before damage = 1:

$$I_{norm}\left(x\right)=\frac{I\left(x\right)}{I_{beforedamage}}$$

For Figures S1C and S1D, the raw intensity values were obtained by manually drawing 10 pixel wide line at the site of damage and the mean value obtained was background subtracted. Similarly, for Figures S1E–S1G, the mean intensity values were recorded at each frame during the time course, background subtracted (*I*_*x*_) and normalized to the mean intensity value before damage to monitor the relative changes in intensity with respect to the intensity before damage:

$$I_{norm}\left(x\right)=\frac{I\left(x\right)}{I_{beforedamage}}\cdot 100$$

All the intensity profiles were aligned at the point immediately after damage as indicated by the vertical dotted lines and depicted as mean and standard deviation of the normalized intensity at each time point.

For Figures 1H and S1H and S1I, the intensity profiles over time were generated by drawing a 10 pixel wide line at the site of damage to obtain mean intensity values at each time point during acquisition which were subsequently background subtracted (*I*_*x*_). All the intensity profiles were aligned to the time point immediately after damage and normalized to the mean intensity value immediately after damage (*I*_*afterdamage*_) (intensity immediately after damage = 1).

$$I_{norm}\left(x\right)=\frac{I\left(x\right)}{I_{afterdamage}}$$

Mean values and their standard deviation at each time point were then calculated from all the pooled intensity profiles depicted in red in Figures S1H and S1I and the values at 4 time points are plotted in Figure S1J. For the plot in Figure 1H, only the initial linear part of tubulin intensity plots in Figures S1H and S1I were used, for tubulin alone, t = 0 to t = 45 s and for CLASP2α, t = 0 to t = 20 s.

#### Intensity analysis for Rhodamine tubulin and CLASP/TOG2-S along a microtubule

Intensity profiles extraction and alignment of Rh-tubulin and CLASP/TOG2-S (Figures 1G and S1B) were performed using custom written MATLAB routine. First, we obtained microtubule average intensity profile along 6-pixel wide line using Rh-tubulin channel. The same line was used to get intensity profile in the CLASP2α/TOG2-S (GFP) channel. After background subtraction each intensity profile I(x) was normalized

$$I_{norm}\left(x\right)=\frac{I\left(x\right)-I_{\min}}{I\mathrm{mi}n_{\max}\cdot 100\%}$$

with respect to the maximum and minimum intensity values along the whole profile. The normalized intensity profiles of different microtubules were aligned so that the plus end tip position was at the origin of the coordinates (Figures 1G and S1B). The plus end position was determined by fitting the Rh-tubulin profile to a Gaussian survival function using equation:

$$I_{norm}\left(x\right)=\frac{1}{2}I_{MT}erfc\left(\frac{x-x_{PF}}{\sqrt{2}\sigma_{PF+PSF}}\right)+I_{BG}$$

where is the complimentary error function, $I_{MT}$ and $I_{BG}$ are average intensities of the microtubule and the background, $x_{PF}$ is the position of plus end tip and $\sigma_{PF+PSF}$ is the standard deviation of the microtubule tip taper combined with the one for the microscope point spread function.

#### Microtubule repair assays with Taxol-stabilized microtubules

Taxol-stabilized microtubules were prepared by polymerizing 29 μM porcine brain tubulin containing 13% biotinylated-tubulin and 6% Rhodamine-labeled tubulin in MRB80 buffer supplemented with 2 mM GTP at 37°C for 30 min. Taxol (Sigma-Aldrich) (18 μM) was then added to the tubulin-GTP mix and seeds were then sedimented by centrifugation at 16,200 × g for 15 min at room temperature. Finally, the pellet was resuspended in warm 10 μM Taxol solution in MRB80 buffer. Taxol-stabilized microtubules were then wrapped with aluminum foil and stored at room temperature for a maximum of 2 weeks.

For tubulin incorporation experiments, Taxol-stabilized microtubules were immobilized in the flow chamber and were washed immediately with the wash buffer (80 mM PIPES, 4 mM MgCl_2_, 1 mM EGTA, 50 mM KCl, 0.5 mg/ml κ-casein, 0.1% methylcellulose, and oxygen scavenger mix (50 mM glucose, 400 μg/ml glucose-oxidase, 200 μg/ml catalase, and 4 mM DTT)). Time-lapse movies were immediately started on the TIRF microscope at 30°C at a 2 s time interval with 100 ms exposure time for 25 minutes. During the imaging session, microtubules were incubated in the washing buffer without Taxol and tubulin for 1.5 min to promote lattice defect formation. Subsequently, they were incubated in MRB80 buffer supplemented with 5 μM HiLyte Fluor 488-labeled tubulin, 50 mM KCl, 1 mM GTP, 0.5 mg/ml κ-casein, 0.1% methylcellulose, and oxygen scavenger mix (50 mM glucose, 400 μg/ml glucose-oxidase, 200 μg/ml catalase, and 4 mM DTT) with or without 30 nM mCherry-CLASP2α or 30 nM mCherry-TOG2-S for 10 min to promote repair. Finally, the residual free green tubulin was washed out with the wash buffer supplemented with 25% glycerol to prevent microtubule depolymerization and to clearly visualize incorporation of green tubulin into the damaged microtubule lattices. In the analysis, defects longer than 1 μm in size, detectable as gaps in the microtubule, where we can clearly see incorporation at both plus and minus ends of the defect site were considered. Partial repair was defined as an event where we did not see a continuous signal of incorporated tubulin in the green channel along the gap at the repair site after the 25% glycerol washing step.

#### Microtubule repair assays with mechanically damaged microtubules

##### Tubulin purification and labeling

For microtubule bending experiments, tubulin was purified from fresh bovine brain by three cycles of temperature-dependent assembly and disassembly in Brinkley Buffer 80 (BRB80 buffer: 80 mM PIPES, pH 6.8, 1 mM EGTA, 1 mM MgCl_2_ plus 1 mM GTP). MAP-free brain tubulin was purified by cation-exchange chromatography (Fractogel EMD SO, 650 M, Merck) in 50 mM PIPES, pH 6.8, supplemented with 1 mM MgCl_2_ and 1 mM EGTA. Purified tubulin was obtained after a cycle of polymerization and depolymerization. Fluorescent tubulin (ATTO-565-labeled tubulin) and biotinylated tubulin were prepared as follows: Microtubules were polymerized from brain tubulin at 37°C for 30 min and layered onto cushions of 0.1 M Na-HEPES, pH 8.6, 1 mM MgCl_2_, 1 mM EGTA, 60% v/v glycerol, and sedimented by high-speed centrifugation at 30°C. Then, microtubules were resuspended in 0.1 M Na-HEPES, pH 8.6, 1 mM NHS-ATTO (ATTO Tec), or NHS-Biotin (Pierce) for 10 min at 37°C. The labeling reaction was stopped using 2 volumes of 2x BRB80, containing 100 mM potassium glutamate and 40% v/v glycerol, and then microtubules were sedimented onto cushions of BRB80 supplemented with 60% glycerol. Microtubules were resuspended in cold BRB80. Microtubules were then depolymerized and a second cycle of polymerization and depolymerization was performed before use.

##### Cover glass micropatterning

The micropatterning technique was adapted from [28]. Cover glasses were cleaned by successive chemical treatments: 30 min in acetone, 15 min in ethanol (96%), rinsing in ultrapure water, 2 h in Hellmanex III (2% in water, Hellmanex), and rinsing in ultrapure water. Cover glasses were dried using nitrogen gas flow and incubated for three days in a solution of tri-ethoxy-silane-PEG (30 kDa, PSB-2014, Creative PEGWorks) 1 mg/ml in ethanol (96%) and 0.02% HCl, with gentle agitation at room temperature. Cover glasses were then successively washed in ethanol and ultrapure water, dried with nitrogen gas, and stored at 4°C. Passivated cover glasses were placed into contact with a photomask (Toppan) with a custom-made vacuum-compatible holder and exposed to deep UV (7 mW/cm^2^ at 184 nm, Jelight) for 2.5 min. Deep UV exposure through the transparent micropatterns on the photomask created oxidized micropatterned regions on the PEG-coated cover glasses.

##### Microfluidic circuit fabrication and flow control

The microfluidic device was fabricated in polydimethylsiloxane (PDMS, Sylgard 184, Dow Corning) using standard photolithography and soft lithography. The master mold was fabricated by patterning 85 μm thick negative photoresist (SU8 3050, Microchem, MA) by photolithography. A positive replica was fabricated by replica molding PDMS against the master. Prior to molding, the master mold was silanized with trichloro(1H,1H,2H,2H-perfluorooctyl)silane (Sigma) for easier lift-off. Four inlet and outlet ports were made in the PDMS device using 0.5 mm soft substrate punches (UniCore 0.5, Ted Pella, Redding, CA). The PDMS device was then brought into contact with a micropatterned cover glass and placed in a custom-made holder that could be fitted on the microscope stage. A transparent plate was fixed on the holder to apply gentle pressure on the chip in order to avoid leaks without the need of permanent bonding to the cover glass. The top plate had four openings for the inlet and outlet tubing. Teflon tubing (Tefzel, inner diameter: 0.03’’, outer diameter: 1/16’’, Upchurch Scientific) was inserted into the two ports serving as outlets. Tubing with 0.01’’ inner and 1/16’’ outer diameter was used to connect the inlets via two three-way valves (Omnifit Labware, Cambridge, UK) that could be opened and closed by hand to a computer-controlled microfluidic pump (MFCS-4C, Fluigent, Villejuif, France). Flow inside the chip was controlled using the MFCS-Flex control software (Fluigent). Custom rubber pieces that fit onto the tubing were used to close the open ends of the outlet tubing when needed.

##### Microtubule growth on micropatterns

Microtubule seeds were prepared at 10 μM tubulin concentration (30% ATTO-565 or ATTO-488-labeled tubulin and 70% biotinylated tubulin) in BRB80 supplemented with 0.5 mM GMPCPP at 37°C for 1 h. The seeds were incubated with 1 μM Taxotere (Sigma) at room temperature for 30 min and were then sedimented by high speed centrifugation at 30°C and resuspended in BRB80 supplemented with 0.5 mM GMPCPP and 1 μM Taxotere. Seeds were stored in liquid nitrogen and quickly warmed to 37°C before use.

The holder with the chip was fixed on the stage and the chip was perfused with NeutrAvidin (25 μg/ml in BRB80, Pierce), then washed with BRB80, passivated for 20 s with PLL-g-PEG (Pll 20K-G35-PEG2K, Jenkam Technology) at 0.1 mg/ml in 10 mM Na-HEPES (pH 7.4), and washed again with BRB80. Microtubule seeds were flown into the chamber at high flow rates perpendicular to the micropatterned lines to ensure proper orientation of the seeds. Unattached seeds were washed out immediately using BRB80 supplemented with 1% BSA. Seeds were elongated with a mixture containing 27 μM tubulin (20% labeled) in BRB80 supplemented with 50 mM NaCl, 25 mM NaPi, 1 mM GTP, an oxygen scavenger cocktail (20 mM DTT, 1.2 mg/ml glucose, 8 μg/ml catalase and 40 μg/ml glucose oxidase), 0.1% BSA and 0.033% methyl cellulose (1500 cp, Sigma). Microtubules were bent by an orthogonal fluid flow either using the same mixture supplemented with 0.02% red fluorescent beads (0.52 μm diameter, Thermo Scientific) or supplementing it additionally with 30 nM GFP-CLASP2α.

##### Imaging

Microtubules were visualized using an objective-based azimuthal ILAS2 TIRF microscope (Nikon Eclipse Ti, modified by Roper Scientific) and an Evolve 512 camera (Photometrics). The microscope stage was kept at 35°C using a warm stage controller (LINKAM MC60). Excitation was achieved using 491 and 561 nm lasers (Optical Insights). Time-lapse recording was performed using Metamorph software (version 7.7.5, Universal Imaging). Movies were processed to improve the signal/noise ratio (smooth and subtract background functions of ImageJ, version 2.2.0-rc-65 / 1.51 s).

##### Measurement of microtubule persistence length

The microtubule is described as an inextensible slender filament with length L and bending rigidity κ, which is bent in two dimensions by the fluid flow. Its elastic energy E is given by

Equation 1 $$E\left(r\right)=\int_{0}^{L}\left\{\frac{k}{2}{\left(\frac{d^{2}r}{ds^{2}}\right)}^{2}+\frac{\lambda }{2}\left[{\left(\frac{dr}{ds}\right)}^{2}-1\right]\right\}ds$$

The vector **r**(s) denotes the position of the filament parameterized by the arc length s and λ denotes a Lagrange multiplier associated with the inextensibility condition |d**r**/ds| = 1. The force exerted on the filament is given by the functional variation of the potential E with respect to the filament position vector **r**

Equation 2 $$F_{B}=-\frac{\delta E}{\delta r}$$

The filament orientation is fixed by the seed orientation at s = 0, whereas the other end of the filament at s = L is force-free. The hydrodynamic drag exerted by the fluid flow on a slender filament is given by

Equation 3 $$F_{H}=g\mu \left(I-\frac{1}{2}\frac{dr}{ds}⊗\frac{dr}{ds}\right)v_{b}$$

where **v**_b_ denotes the velocity field measured by the bead displacements, μ denotes the viscosity of the fluid and g denotes a geometrical factor of the order of 1, which depends on the distance of the filament from the surface, the radius of the filament and the distance of the beads from the surface. ⊗ denotes the outer product and *I* is the identity tensor. In mechanical equilibrium

Equation 4 $$F_{B}+F_{H}=0$$

which determines the equilibrium shape of the filament subject to the appropriate boundary conditions. The filament rigidity was determined by solving Equations 1, 2, 3, and 4 using the AUTO-07p software package and by minimizing the function

Equation 5 $$\omega^{2}\left(\kappa \right)=\frac{1}{L}\int_{0}^{L}{\left[r_{R}\left(s\right)-r\left(s\right)\right]}^{2}ds\;$$

where **r**_R_(s) denotes the measured position of the filament. The persistence length is then given by L_p_ = κ/(k_B_ T). ω is a measure for the distance between the shapes of two microtubules. In the fitting routine for the experimentally measured microtubule shapes, ω denotes the distance between the shape of the experimental snake and an inextensible flexible filament subjected to the same flow as the experimental snake. We assumed that the origin of the microtubule was clamped in the direction of the seed. To correct for a measurement error of the microtubule origin, we optimized Equation 5 also for the position of the microtubule origin.

#### Mass spectrometry

Purified CLASP2α was run on SDS-PAGE gel. After in-gel digestion, samples were resuspended in 10% formic acid (FA)/5% DMSO and were analyzed using an Agilent 1290 Infinity (Agilent Technologies) LC connected to an Orbitrap Q-Exactive HF mass spectrometer (Thermo Fisher Scientific). Samples were first trapped (Dr Maisch Reprosil C18, 3 um, 2 cm x 100 μm) before being separated on an analytical column (Agilent Poroshell EC-C18, 2.7 μm, 40 cm x 50 μm), using a gradient of 50 min at a column flow of 150 nl/min. The mass spectrometer was used in a data-dependent mode, automatically switching between MS and MS/MS. Full scan MS spectra from m/z 375 – 1600 were acquired at a resolution of 60.000 after the accumulation to a target value of 3E6. HCD fragmentation of up to 15 most intense precursor ions was performed at normalized collision energy of 25% after the accumulation to a target value of 1e5. MS2 was acquired at a resolution of 15.000 and dynamic exclusion was enabled. Raw files were processed using Proteome Discoverer 1.4 (version 1.4.0.288, Thermo Fisher Scientific). The database search was performed using Mascot (version 2.4.1, Matrix Science, UK) against the Uniprot human database (version 2.4). Carbamidomethylation of cysteines was set as a fixed modification and oxidation of methionine was set as a variable modification. Trypsin was specified as enzyme and up to two miss cleavages were allowed. Data filtering was performed using percolator, resulting in 1% false discovery rate (FDR). Additional filters were; search engine rank 1 peptides and ion score > 20.

### Quantification and Statistical Analysis

Kymographs were generated using the ImageJ plugin KymoResliceWide (https://github.com/ekatrukha/KymoResliceWide). MT growth rate was determined from kymographs using an optimized version of the custom-made JAVA plug in for ImageJ as described previously [12]. Number of samples, replicates and error bars have been indicated in the figure legends. Spearman correlation test was performed to test for microtubule softening. All statistical analyses were performed using GraphPad Prism 7 and the details of statistical tests and the resulting p values were included in the figure legends.

### Data and Code Availability

ImageJ plugin KymoResliceWide is available online at https://github.com/ekatrukha/KymoResliceWide. All data supporting the conclusions of the current study are available from the corresponding author on request.

## Supplemental Information

Document S1. Figures S1 and S2

Table S1. Mass Spectrometry Analysis of StrepII-GFP-CLASP2α Purified from HEK293T Cells, Related to Figure 1 The hits with more than 10 peptides are listed.

Document S2. Article plus Supplemental Information
