# Supplementary material for: CLASP Mediates Microtubule Repair by Restricting Lattice Damage and Regulating Tubulin Incorporation
Source: Curr Biol. 2020 Jun 8;30(11):2175–2183.e6. doi: 10.1016/j.cub.2020.03.070 (PMC7280784; doi:10.1016/j.cub.2020.03.070)
Supplement: Document S1. Figures S1 and S2 [file mmc1.pdf]

Current Biology, Volume *30*

## Supplemental Information

# CLASP Mediates Microtubule Repair

# by Restricting Lattice Damage

# and Regulating Tubulin Incorporation

**Amol Aher, Dipti Rai, Laura Schaedel, Jeremie Gaillard, Karin John, Qingyang Liu, Maarten Altelaar, Laurent Blanchoin, Manuel Thery, and Anna Akhmanova**

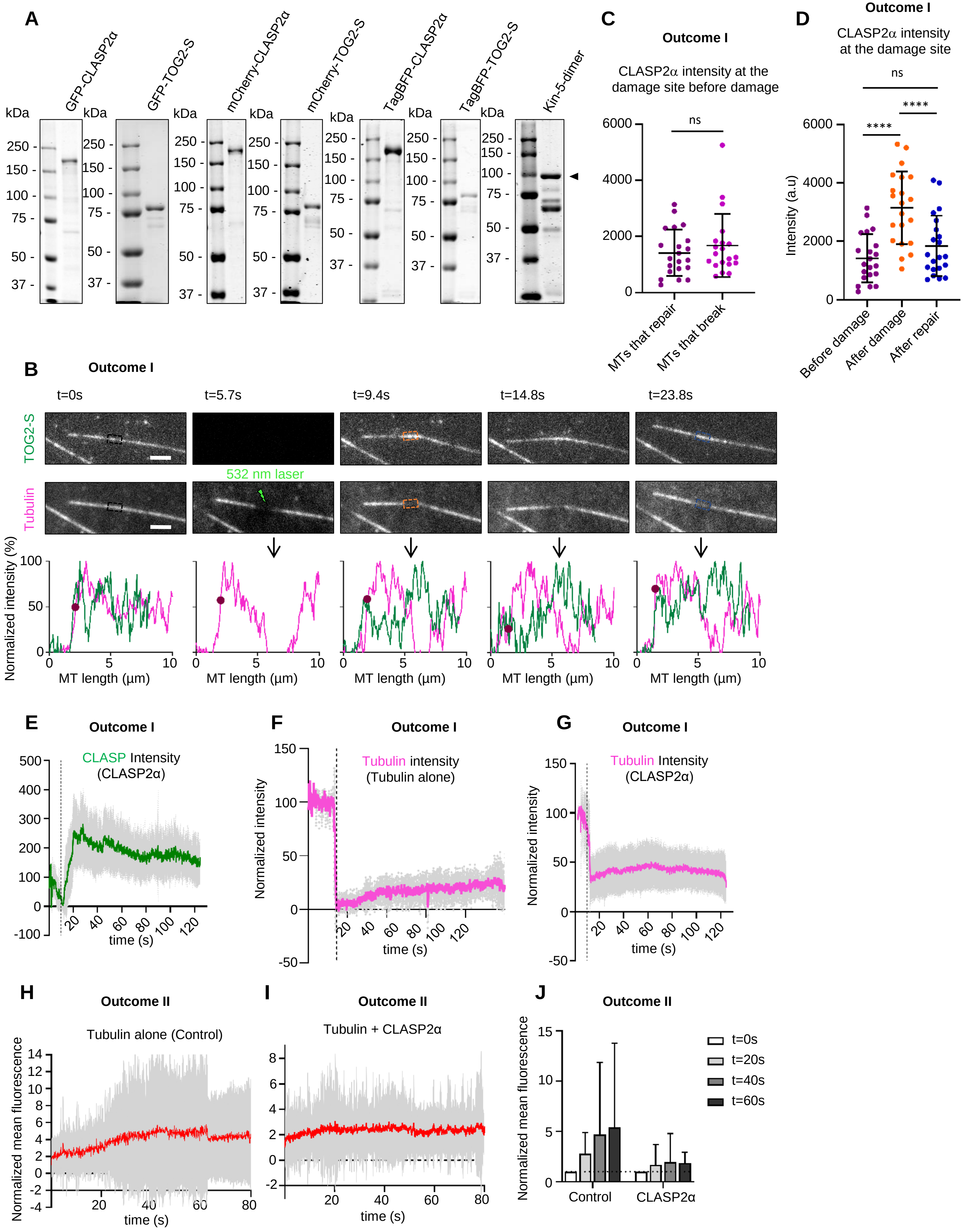


**Figure S1. Characterization of CLASP binding and tubulin incorporation at the sites of microtubule damage, related to Figure 1.**

(A) Proteins purified from HEK293T cells (CLASP2α and TOG2-S constructs) and *E.coli* (Kin-5-dimer) used in this study analyzed by SDS-PAGE.

(B) Stills from a time lapse of a dynamic microtubule grown in the presence of 30 nM GFP-TOG2-S (upper panel) and Rhodamine-tubulin (lower panel) damaged at a point along the lattice as indicated. Intensity profiles along the microtubule for the TOG2-S and tubulin channel at different time points are shown below, with the arrow pointing to the site of photodamage. The purple circle on the plot indicates the end of the microtubule. Scale bar: 2 µm.

(C) Fluorescence intensity of CLASP2α at the irradiated site along the lattice before damage for dynamic microtubules grown in the presence of 30 nM GFP-CLASP2α that were either repaired (n=21 microtubules) or broke (n=20 microtubules). Error bars denote SD.

(D) Fluorescence intensity of CLASP2α at the photo-damage site along the lattice for dynamic microtubules grown in the presence of 30 nM GFP-CLASP2α before damage, after damage and after repair. The same, but normalized data are shown in Figure 1E. Error bars denote SD. n=21 microtubules.

(E) Mean intensity of CLASP2α normalized to the intensity before damage (set at 100) over time at the photodamage site for microtubules that bent to an angle $>10^o$ at the point of photodamage and subsequently straightened (Outcome I). n=21 microtubules.

(F, G) Mean tubulin fluorescence intensity normalized to the intensity before damage (set at 100) over time at the photodamage site for dynamic microtubules grown in the presence of tubulin alone (F) or together with 30 nM GFP-CLASP2α (G). Only microtubules that bent to an angle $>10^o$ at the point of photodamage and subsequently straightened (Outcome I) are included. All the intensity profiles were aligned at the point immediately after damage as indicated by the vertical dotted lines. Tubulin alone: n=4 microtubules; CLASP2α: n=21 microtubules.

(H,I) Mean fluorescence intensity at the photodamage site over time normalized to the intensity immediately after damage (set at 1) for microtubules grown in the presence of Rhodamine-tubulin alone (H) (n=35 microtubules from 2 experiments) or together with 30 nM GFP-CLASP2α (I) (n=44 microtubules from 2 experiments). Red line shows the mean values, grey bars indicate the standard deviation.

(J) Tubulin intensity at the photodamage site after repair with respect to the intensity immediately after damage at the indicated time points for microtubules grown in the presence of Rhodamine-tubulin alone (n=35 microtubules from 2 experiments) or together with 30 nM GFP-CLASP2α (n=44 microtubules from 2 experiments). Error bars represent SD.

For all plots ****p<0.0001, ***p=0.001 and ns=no significant difference with control, Mann Whitney U test.

See also Video S1 and S2.

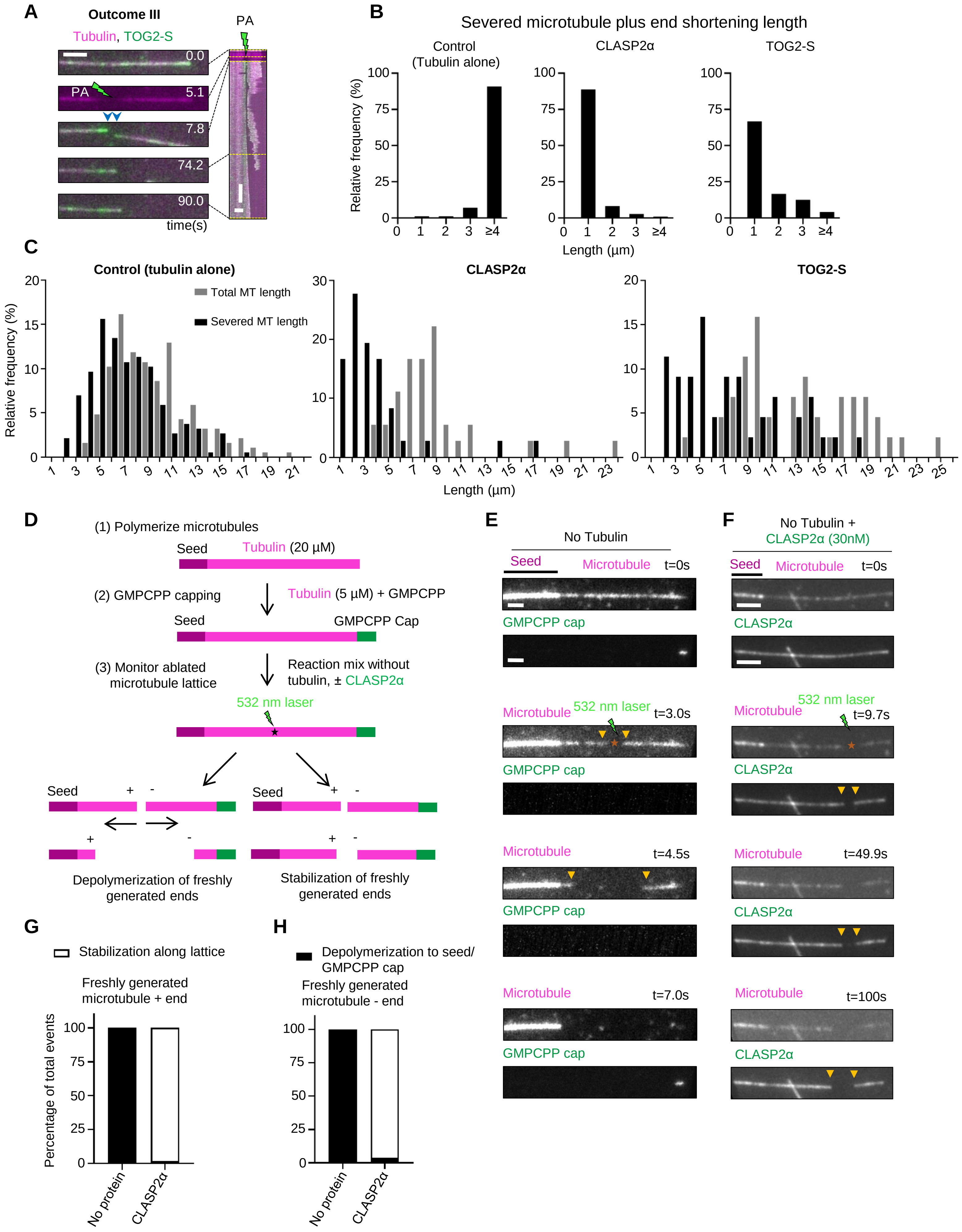


**Figure S2. CLASP protects severed microtubules from depolymerization, related to Figure 1.**

(A) Stills and the corresponding kymograph of a microtubule grown in the presence of Rhodamine-tubulin together with GFP-TOG2-S (30 nM) ablated with a 532 nm laser as indicated. Scale bars: still image, 2 µm; kymograph, 4 µm (horizontal) and 10 sec (vertical). Dotted yellow lines point to the time point of the still in the kymograph.

(B) Plots showing the relative frequencies of microtubule plus end shortening lengths after laser-mediated severing of dynamic microtubules grown in the presence of Rhodamine-tubulin alone or together with either 30 nM GFP-CLASP2α or with 30 nM TOG2-S. n=186 microtubules analyzed from 3 experiments for tubulin alone, n=36 microtubules analyzed from 3 experiments for CLASP2α and n=48 microtubules analyzed from 8 experiments for TOG2-S.

(C) Plot showing the relative frequencies of total microtubule lengths (grey) and severed microtubule lengths (black) for microtubules grown in the presence of Rhodamine-tubulin alone (left panel) or together with either 30 nM GFP-CLASP2α (middle panel) or with 30 nM GFP--TOG2-S (right panel). n=186 microtubules analyzed from 3 experiments for tubulin alone, n=36 microtubules analyzed from 3 experiments for CLASP2α and n=48 microtubules analyzed from 8 experiments for TOG2-S.

(D) Schematic for an experiment to monitor microtubule lattice damage in the absence of tubulin. Microtubules were polymerized in the presence of Rhodamine tubulin followed by capping with GMPCPP. Tubulin was then washed out and microtubules were ablated along the lattice either in the presence of reaction mixture alone or together with 30 nM GFP-CLASP2α.

(E,F) Stills from a time lapse showing microtubules and GMPCPP cap/ CLASP2α and GMPCPP cap at different time points. Yellow arrowheads indicate the position of the freshly generated ends upon photoablation. Scale bar: 2 µm.

(G,H) Plot showing the outcome for freshly generated plus end (G) and minus end (H) of a microtubule upon laser induced photo-ablation in the presence of reaction mixture without tubulin (no protein) alone (n=29 microtubules from 2 experiments) or together with 30 nM GFP-CLASP2α (n=56 from 2 experiments).
